# Supplementary material for: Eukaryotic Microalgae Communities from Tropical Karstic Freshwater Lagoons in an Anthropic Disturbance Gradient Microscopic and Metagenomic Analysis
Source: Microorganisms. 2024 Nov 20;12(11):2368. doi: 10.3390/microorganisms12112368 (PMC11596289; doi:10.3390/microorganisms12112368)
Supplement: Supplementary file 1 [file microorganisms-12-02368-s001.zip › microorganisms-3251363-supplementary-author.pdf]

Supplementary Files SF1. Incidence Table of Taxa

Genus	MDS	MCS	MCP	XDS	XBS	SDS	SCS	SCP	SBS
Anabaena	0	0	1	1	0	1	0	0	0
Ankistrodesmus	0	0	0	0	0	1	1	1	1
Arthrospira	0	0	0	0	0	1	1	1	0
Asterocapsa	1	0	0	0	1	1	0	0	0
Caloneis	0	0	0	0	0	0	0	1	0
Chlorella	1	0	0	0	0	1	0	0	0
Chlorococcum	1	0	0	0	0	1	0	0	0
Chroococcus	1	0	0	0	0	1	0	1	0
Chlamydomona	1	0	0	0	1	1	0	1	0
Cocconeis	1	0	0	0	0	0	0	0	0
Coelastrella	1	0	0	0	1	0	0	0	0
Coelastrum	1	0	1	0	0	1	0	0	0
Coscinodiscus	1	0	0	0	0	0	0	0	0
Cosmarium	1	1	1	1	1	0	1	1	0
Crucigenia	0	0	0	0	0	0	0	1	0
Cymbella	0	0	0	0	0	1	0	0	0
Desmodesmus	0	0	0	0	0	1	0	0	0
Diatoma	0	0	0	0	0	1	0	0	0
Dictyococus	1	0	0	0	1	1	0	0	0
Euglena	0	0	0	0	0	1	0	0	1
Frustulia	0	0	1	0	0	0	0	0	0
Glaucocystis	0	0	0	0	0	1	0	0	0
Glennodinium	1	0	0	0	0	0	0	0	0
Gloeocystis	1	0	0	0	0	1	0	0	0
Gomphonema	0	0	0	0	0	1	0	0	0
Gomphosphaeria	1	0	0	0	1	1	0	0	0
Gymnodinium	1	0	0	0	0	0	0	0	0
Hassallia	0	0	0	0	0	1	0	0	0
Klebsormidium	0	0	0	0	0	1	0	0	1
Lepocinclis	0	0	0	0	0	1	0	0	0
Leptolyngbya	1	0	0	0	1	1	1	0	0
Limnothrix	0	0	0	0	0	1	0	0	0
Lyngbya	1	0	0	0	0	0	0	0	0
Merismopedia	0	0	0	0	1	1	0	1	0
Mesotaenium	0	0	0	0	0	1	0	0	0
Microcoleus	1	0	0	0	0	1	0	0	0
Navicula	1	0	0	0	0	1	0	0	0
Neochloris	1	0	0	0	0	0	0	0	0
Nitzschia	0	0	1	0	0	1	0	0	0
Nostoc	0	0	0	0	0	1	0	0	0
Oocystis	1	0	0	0	0	1	1	1	0
Oscillatoria	1	0	0	0	0	0	0	0	0
Pandorina	1	0	1	0	0	1	0	1	0

Pediastrum	0	0	0	0	0	1	0	0	0
Peridinium	1	0	0	0	0	0	0	0	0
Phacus	1	0	1	0	0	1	0	1	0
Phormidium	1	0	0	0	0	1	0	0	0
Pinnularia	0	0	0	1	0	0	0	0	0
Pleurococcus	1	0	0	0	0	0	0	0	0
Pseudanabaena	1	0	0	0	0	0	0	0	0
Rhizoclonium	1	0	0	0	0	0	0	0	0
Scenedesmus	0	0	0	0	0	1	0	0	0
Scrippsiella	0	0	1	0	0	0	0	0	0
Scytonema	0	0	0	0	0	0	0	1	0
Sellaphora	0	0	0	0	0	1	0	0	0
Staurastrum	0	0	0	0	0	1	0	0	0
Stauroneis	0	0	0	0	0	1	0	0	0
Surirella	0	0	0	1	0	1	0	1	0
Tetraedron	1	1	1	0	0	1	0	1	1
Tribonema	0	0	0	0	0	1	0	0	0
Ulnaria	0	0	0	1	0	1	0	0	0
Vischeria	1	0	0	0	1	0	0	0	0

Supplementary File SF2. Diversity Indices and absolute abundance Table

	MDS	MCS	MCP	XDS	XBS	SDS	SBS	SCS	SCP
Taxa_S	211	253	182	125	160	283	98	376	122
Individuals	463	532	407	223	329	569	173	746	424
Dominance_D	0.01	0.008	0.013	0.009	0.011	0.008	0.011	0.006	0.03
Simpson_1-D	0.99	0.992	0.987	0.991	0.989	0.992	0.989	0.994	0.97
Shannon_H	5.122	5.362	4.941	4.85	4.937	5.437	4.628	5.738	4.246
Evenness_e^H/S	0.795	0.843	0.769	1.022	0.871	0.812	1.044	0.826	0.572
Brillouin	4.358	4.565	4.192	3.936	4.129	4.615	3.708	4.904	3.733
Menhinick	9.806	10.97	9.021	8.371	8.821	11.86	7.451	13.77	5.925
Margalef	34.21	40.15	30.12	22.93	27.43	44.45	18.82	56.69	20
Equitability_J	0.957	0.969	0.95	1.005	0.973	0.963	1.009	0.968	0.884
Fisher_alpha	149.8	188.9	126.4	117.5	122.9	223.7	93.67	302.5	57.34
Berger-Parker	0.054	0.045	0.047	0.036	0.043	0.044	0.04	0.035	0.116
Chao-1	589.8	595.1	478.8	384.9	463.2	722.1	227.2	1028	198.1
iChao-1	650.7	671.6	545	434.3	522.4	791.3	256.8	1141	222.6
ACE	572.1	629.5	459.9	325.8	450.1	835.4	233	1100	214.2
Squares	501	560.1	435.8	253.8	349.9	743.3	187.8	987.7	248.9
Genus	MDS	MCS	MCP	XDS	XBS	SDS	SBS	SCS	SCP
Euglenophyceae	9	7	11	0	7	25	1	17	0
Symbiodinium	12	11	10	8	9	9	7	10	0
Theileria	4	4	3	1	3	3	2	4	49
Desmodesmus	10	10	0	4	14	14	4	15	0
Chlorella	9	8	0	8	8	10	7	15	0
Hydrodictyon	13	11	0	3	4	16	2	14	0
Volvocaceae	8	9	0	6	10	8	4	13	0
Nannochloropsis	6	6	11	8	7	6	0	7	0
Cryptomonas	7	7	5	0	3	7	1	9	8
Ankistrodesmus	4	5	0	5	7	8	5	8	0
Acytosteliaceae	6	7	6	4	4	4	4	4	1
Eustigmatophyceae sp.	6	5	5	4	5	6	1	6	0
Micromonas	3	3	4	3	3	4	2	4	9
Vischeria	4	3	6	2	2	5	0	5	0
Auxenochlorella	4	3	1	3	4	4	2	4	0
Chroomonas	6	5	2	1	1	3	0	3	4
Synura	5	4	0	0	3	6	0	6	0
Ostreococcus	3	3	3	3	3	3	2	3	0
Pseudochloris	1	1	19	0	0	1	0	1	0
Ptilothamnion	0	0	1	0	0	0	0	0	21
Coccomyxa	3	4	0	3	3	3	2	3	0
Diacronema	3	3	3	1	3	2	1	4	1
Trebouxia	2	2	6	3	1	4	1	2	0
Ectocarpus	3	3	0	3	3	3	2	3	0
Porphyridium	1	2	3	1	1	3	0	2	6
Thraustotheca	1	1	1	0	1	1	1	1	12

Chloropicon	3	2	2	1	2	1	1	6	0
Cyanophora	3	2	2	0	0	4	0	3	4
Prorocentrum	4	5	2	0	1	3	0	3	0
Dunaliellaceae	2	4	0	2	3	2	1	3	0
Emiliania	2	2	2	1	1	2	1	1	5
Guillardia	2	2	2	1	2	2	1	2	3
Alexandrium	3	4	3	0	0	1	0	4	0
Microchloropsis	2	3	0	2	2	2	2	2	0
Amphidinium	2	4	2	0	0	2	0	2	2
Cyanidiococcus	2	2	2	2	2	1	1	2	0
Nephroselmis	1	2	3	1	0	2	0	2	3
Bodo	2	2	1	1	1	2	2	2	0
Glaucocystis	1	1	2	0	0	2	1	3	3
Gracilariopsis	1	2	2	1	2	2	1	2	0
Haramonas	0	1	0	0	0	0	0	0	12
Ochromonas	2	3	0	1	1	2	0	4	0
Ulva	0	1	5	2	2	1	0	2	0
Ahnfeltia	1	2	1	1	1	1	0	3	2
Carpediemonas	2	1	1	2	1	2	0	1	2
Globisporangium	1	3	3	1	1	1	1	1	0
Proteomonas	1	3	0	1	1	2	0	2	2
Thecamonas	1	2	1	1	1	1	1	2	2
Vitrella	2	2	1	1	1	1	1	1	2
Chlamydomonadales in	2	1	0	1	1	2	1	3	0
Chrysochromulina	1	1	1	1	1	2	1	3	0
Cyanidiaceae	1	0	1	0	0	0	0	1	8
Neocystis	2	3	0	0	1	3	0	2	0
Oedogonium	0	2	2	0	1	1	0	5	0
Pelagomonas	1	2	1	1	1	2	1	2	0
Quadricoccopsis	1	2	3	0	1	1	0	3	0
Reclinomonas	1	0	1	0	1	1	0	2	5
Tetraedron	0	2	0	1	1	2	0	5	0
Chattonella	1	2	0	0	0	2	0	5	0
Choricystis	1	1	0	1	2	2	1	2	0
Cyanidioschyzon	1	1	2	0	1	2	1	2	0
Helicosporidium	2	2	0	1	2	1	0	2	0
Heterocapsa	2	3	2	1	0	1	0	1	0
Histiona	3	1	1	1	0	0	1	2	1
Kappaphycus	0	1	0	0	0	0	0	0	9
Perkinsela	1	2	1	0	1	1	1	1	2
Botryococcus	1	1	0	1	1	3	0	2	0
Bracteacoccus	0	1	0	0	1	4	0	3	0
Dictyopteris	1	0	0	0	0	4	0	4	0
Elliptochloris	0	0	7	0	0	1	0	1	0

Haematococcaceae	2	1	0	1	1	1	1	2	0
Hepatocystis	1	1	0	1	2	2	1	1	0
Histomonas	1	1	1	1	1	1	0	1	2
Mallomonas	1	2	0	0	0	3	0	3	0
Melanthalia	0	0	0	0	0	0	0	1	8
Melobesioideae	2	2	1	1	1	1	0	1	0
Pedinomonas	1	2	1	0	1	0	0	1	3
Pedinophyceae sp.	1	1	1	1	1	1	1	1	1
Pelomyxidae	1	1	1	1	1	1	1	1	1
Planoprotostelium	1	1	1	1	1	1	1	1	1
Porphyra	1	1	1	1	1	2	1	1	0
Pycnococcus	1	1	1	1	1	1	1	1	1
Rhodomonas	1	2	0	0	0	2	0	3	1
Trebouxiophyceae sp.	0	1	2	1	1	2	0	2	0
Trichomonas	1	1	1	1	1	1	0	1	2
Achlya	1	1	1	1	1	1	1	1	0
Agarophyton	1	1	0	0	2	2	1	1	0
Andalucia	1	0	1	0	1	1	1	1	2
Aureococcus	1	1	1	1	1	1	1	1	0
Bathycoccus	1	1	1	1	1	1	1	1	0
Characiopsis	2	2	0	0	1	2	0	1	0
Colpomenia	0	0	4	0	0	1	0	3	0
Eremosphaera	2	1	0	0	2	1	0	2	0
Galdieria	1	1	1	1	1	1	1	1	0
Jenufa	0	0	3	0	0	2	0	3	0
Koliella	2	1	1	0	1	1	0	2	0
Mastigamoebidae	0	0	0	0	0	1	0	1	6
Monomastix	1	1	1	0	1	1	0	2	1
Nusuttodinium	1	2	3	0	0	1	0	1	0
Peronospora	1	1	1	1	2	1	0	1	0
Pfiesteria	2	2	2	0	0	1	0	1	0
Plasmopara	1	1	1	1	1	1	1	1	0
Porolithon	0	0	0	0	0	1	0	1	6
Raperosteliaceae	1	1	1	1	1	1	1	1	0
Scinaia	1	0	0	0	0	0	0	1	6
Streblomastix	1	1	1	1	1	1	1	1	0
Tetrabaenaceae	2	1	0	1	1	1	1	1	0
Thaumatomonas	0	0	0	0	0	0	0	2	6
Thorea	1	1	1	0	1	1	0	1	2
Tritrichomonas	1	2	1	0	0	1	1	1	1
Wildemania	0	0	0	0	0	0	0	1	7
Cafeteria	1	1	0	1	1	1	1	1	0
Cavenderiaceae	1	1	1	0	1	1	1	1	0
Chloroparvula	0	1	2	0	0	3	0	1	0

Chondrus	1	1	1	0	1	1	1	1	0
Giardia	1	1	1	0	0	1	1	2	0
Gracilaria	0	0	0	1	0	1	2	3	0
Gregarina	1	1	0	1	1	1	1	1	0
Herpetomonas	1	1	0	0	0	2	1	2	0
Hondaea	1	1	0	1	1	1	1	1	0
Kipferlia	1	1	1	0	0	1	1	1	1
Nothophytophthora	1	1	1	1	1	1	0	1	0
Ochrosphaera	0	1	1	0	0	1	0	2	2
Peridinium	1	0	2	0	0	1	0	3	0
Plasmodiophora	1	1	0	1	1	1	1	1	0
Polarella	1	1	1	1	1	1	0	1	0
Pseudellipsoidion	1	1	1	0	1	1	0	2	0
Reticulomyxa	1	1	0	1	1	1	1	1	0
Spumella	0	1	0	0	2	2	0	2	0
Stygiella	1	1	1	1	1	0	0	1	1
Trachydiscus	1	1	1	1	1	1	0	1	0
unc.	1	1	1	1	0	1	0	1	1
Vannella	0	0	0	0	0	2	0	1	4
Amoeba	1	1	0	0	0	0	0	1	3
Azadinium	2	2	2	0	0	0	0	0	0
Bulboplastis	1	1	0	1	0	1	0	1	1
Cardiosporidium	1	1	1	0	1	1	0	1	0
Corallinoideae	0	1	1	0	0	2	0	2	0
Erythrotrichia	0	3	1	0	0	1	0	1	0
Gloiopeltis	1	1	1	0	1	1	0	1	0
Goniomonas	0	1	0	0	0	2	0	2	1
Jakoba	1	1	0	0	0	0	0	2	2
Marophrys	0	1	1	0	0	0	1	1	2
Microspora	0	1	0	1	1	2	0	1	0
Monocercomonoides	1	1	1	0	1	1	0	1	0
Monodopsis	1	1	0	1	1	1	0	1	0
Paramoeba	0	0	0	0	0	2	0	1	3
Prasinococcus	0	1	1	0	0	1	0	2	1
Sargassococcus	0	1	0	0	0	0	0	0	5
Scherffelia	1	1	0	1	1	1	0	1	0
Schimmelmannia	1	1	1	0	1	1	0	1	0
Schizocladia	1	1	1	0	0	1	0	1	1
Spironucleus	0	0	1	0	0	0	0	1	4
Telonemida	1	1	1	0	0	1	0	1	1
Tripos	1	1	2	0	0	1	0	1	0
Tsukubamonas	1	1	0	0	0	1	1	1	1
Tsunamia	0	1	1	0	0	1	0	1	2
Vacuolaria	1	1	2	0	0	1	0	1	0

Aureoumbra	0	1	1	1	0	1	0	1	0
Bigelowiella	1	0	0	1	1	0	1	1	0
Chromochloris	0	1	0	1	1	1	0	1	0
Compsopogon	0	1	1	1	1	0	0	0	1
Crouania	1	2	0	0	0	1	0	1	0
Cyanoptyche	1	1	1	0	0	0	0	1	1
Durinskia	1	1	1	0	0	1	0	1	0
Gloeochaete	0	1	1	0	0	1	0	1	1
Heterosigma	1	1	0	1	0	1	0	1	0
Hildenbrandia	0	1	0	0	0	1	0	2	1
Ignatiu	0	1	1	0	1	1	0	0	1
Karenia	0	2	2	0	0	0	0	1	0
Korotnevella	1	1	0	0	0	1	0	1	1
Kuetzingia	1	1	1	0	1	0	0	1	0
Lingulodinium	1	1	1	0	0	1	0	1	0
Lobosphaera	1	0	0	1	1	1	0	1	0
Microthamnion	0	1	2	0	0	1	0	1	0
Myrmecia	1	1	0	0	1	1	0	1	0
Olisthodiscus	0	1	1	0	1	1	0	1	0
Pedospumella	0	1	0	0	1	1	0	2	0
Picocystis	1	0	1	0	0	1	0	1	1
Pleurastraceae	1	1	0	0	1	1	0	1	0
prasinophyte sp.	0	1	1	0	0	1	0	1	1
Prasiola	1	1	0	0	1	1	0	1	0
Prototheca	0	2	0	0	0	2	0	1	0
Prymnesium	0	0	0	0	1	0	0	1	3
Pseudochlorella	1	1	0	0	1	1	0	1	0
Pseudopedinella	0	2	1	0	1	0	0	1	0
Pyramimonas	0	0	1	0	1	0	0	1	2
Pyrocystis	1	1	1	0	0	1	0	1	0
Renouxia	1	1	1	0	0	1	0	1	0
Rhodochaete	0	1	1	0	0	1	0	1	1
Spongospora	0	0	1	0	0	1	0	1	2
Stichogloea	0	1	1	0	0	0	0	0	3
Storeatula	1	0	1	0	0	1	0	1	1
Tetraselmis	0	0	1	1	1	1	0	1	0
Triparma	1	1	0	0	1	1	0	1	0
Uroglenopsis	0	1	4	0	0	0	0	0	0
Uronema	1	1	2	0	0	1	0	0	0
Acetabularia	0	2	0	0	1	0	0	1	0
Apusomonas	1	1	1	0	0	0	0	0	1
Balamuthiidae	0	0	0	0	0	0	0	1	3
Balbiania	1	1	0	0	0	1	0	1	0
Boldia	0	1	1	0	0	0	0	1	1

Chlorosarcina	0	1	0	0	0	1	0	2	0
Chordaria	0	0	0	0	1	1	0	2	0
Chromera	1	1	1	0	0	0	0	1	0
Cladocopium	1	1	1	0	0	0	0	1	0
Crypthecodinium	1	1	1	0	0	0	0	1	0
Cryptocaryon	0	1	1	0	0	1	0	1	0
Cyanidium	0	1	1	0	0	0	0	2	0
Dictyochloris	1	1	0	0	0	1	0	1	0
Dinophysis	1	1	1	0	0	0	0	1	0
Fusochloris	0	0	0	0	1	1	0	2	0
Gelidium	0	0	1	0	0	1	0	2	0
Gonyostomum	1	1	0	0	0	1	0	1	0
Grateloupia	0	0	0	1	0	1	0	1	1
Hemiarma	0	1	0	0	0	1	0	1	1
Karlodinium	1	1	1	0	0	0	0	1	0
Leucocryptos	1	0	1	0	0	1	0	0	1
Malawimonas	0	0	0	0	0	0	0	2	2
Marvania	1	0	0	0	1	1	0	1	0
Moramonas	0	1	1	0	0	0	0	1	1
Neopyropia	0	1	1	0	0	1	0	1	0
Oltmannsiellopsis	1	0	0	1	1	0	0	1	0
Ovalopodium	0	0	0	0	0	0	0	1	3
Palpitomonas	0	1	0	0	0	1	0	1	1
Parachlorella	1	1	0	0	0	1	0	1	0
Paratrypanosoma	1	1	0	1	0	0	1	0	0
Pavlova	0	0	0	0	0	0	0	1	3
Placidia	1	1	0	0	1	0	0	1	0
Pleurostomum	1	0	0	1	0	1	0	0	1
Plocamium	0	0	0	0	1	0	0	1	2
Polysiphonia	0	1	0	0	0	1	0	2	0
Poterioochromonas	1	1	0	0	0	1	0	1	0
Poteriospumella	0	2	1	0	0	0	0	1	0
Protoceratium	1	1	1	0	0	0	0	1	0
Protodinium	1	1	1	0	0	1	0	0	0
Protosteliaceae	1	0	0	0	0	1	0	1	1
Pseudoerythrocladia	0	0	0	0	0	0	0	1	3
Pseudomuriella	0	1	0	0	1	1	0	1	0
Pterocladia	0	0	0	0	0	0	1	1	2
Symbiochloris	0	1	0	1	0	1	0	1	0
Teleaulax	0	1	1	0	0	0	0	1	1
unclassified Chlamydom	1	1	0	0	0	1	0	1	0
unclassified Cryptophyt	0	0	0	0	1	1	0	1	1
Vermamoeba	0	1	1	0	0	0	1	0	1
Acrosorium	0	1	1	0	0	0	0	1	0

Adenoides	1	0	0	0	0	1	0	1	0
Akashiwo	1	1	1	0	0	0	0	0	0
Andersenia	0	2	0	0	0	0	0	1	0
Apophlaea	0	1	1	1	0	0	0	0	0
Asparagopsis	0	1	0	0	0	1	0	1	0
Blepharisma	1	0	0	0	0	2	0	0	0
Bostrychia	0	2	0	0	0	0	0	1	0
Breviolum	1	1	1	0	0	0	0	0	0
Cafileria marina	0	0	0	0	0	1	1	1	0
Caloglossa	0	0	0	0	0	1	0	2	0
Chondria	0	1	0	0	0	1	0	1	0
Chroodactylon	1	0	1	0	0	1	0	0	0
Corynoplastis	0	1	0	0	0	0	0	1	1
Desmarestia	0	0	1	0	0	1	0	1	0
Dicranema	0	0	1	1	0	0	0	1	0
Edaphochlorella	1	0	0	0	0	1	0	1	0
Euglypha	0	1	0	0	0	0	1	1	0
Feldmannia	0	0	0	0	0	0	0	3	0
Flintiella	1	1	1	0	0	0	0	0	0
Gambierdiscus	0	0	1	0	0	1	0	1	0
Geminella	1	0	0	1	0	0	0	1	0
Gloeotilopsis	0	0	0	0	1	1	0	1	0
Halochlorococcum	1	0	1	0	0	0	0	1	0
Haptophyceae sp. NIES-	0	1	0	1	0	0	0	0	1
Hemiselmis	0	0	1	0	0	0	0	1	1
Isochrysis	1	1	0	1	0	0	0	0	0
Jaagichlorella	0	1	0	0	1	1	0	0	0
Kryptoperidinium	1	1	1	0	0	0	0	0	0
Lacrimia	0	1	0	1	0	0	0	0	1
Leptophrys	0	0	0	0	1	1	0	0	1
Lithomelissa	1	1	0	0	0	0	0	1	0
Madagascaria	0	1	1	0	0	0	0	1	0
Marsupiomonas	0	0	0	0	0	1	0	1	1
Martensia	1	1	1	0	0	0	0	0	0
Mesophyllum	0	0	0	0	0	0	0	1	2
Neodangemannia	1	0	0	0	0	1	0	1	0
Neotessella	0	1	0	0	0	1	0	1	0
Nephromyces	1	0	0	1	0	0	0	1	0
Palmaria	0	0	1	0	0	0	0	1	1
Paracercomonas	0	0	0	0	0	1	1	1	0
Paravannella	0	1	1	0	0	0	0	1	0
Parietochloris	0	0	0	0	1	1	0	1	0
Pelagodinium	1	1	1	0	0	0	0	0	0
Peridiniopsis	1	1	1	0	0	0	0	0	0

Phalacroma	1	0	0	0	0	0	0	0	2
Phalansterium	0	1	0	0	0	0	0	1	1
Picozoa	0	0	0	0	1	0	0	1	1
Polypodochrysis	0	0	0	0	0	1	0	0	2
Prasiolopsis	1	0	1	0	0	1	0	0	0
Rhizochromulina	0	0	1	0	0	1	0	1	0
Rotundella	1	0	0	0	0	1	0	1	0
Scrippsiella	0	3	0	0	0	0	0	0	0
Seculamonas	0	1	1	0	0	0	0	1	0
Sticholonche	0	1	0	0	0	1	0	1	0
Taenioma	0	0	0	1	0	1	0	1	0
Toxarium	1	0	0	0	0	1	0	1	0
Watanabea	0	0	1	0	1	1	0	0	0
Xylochloris	0	0	1	0	1	1	0	0	0
Acrasis	0	0	0	1	1	0	0	0	0
Acrochaetium	0	0	1	0	0	1	0	0	0
Aphanochaete	1	0	0	0	0	0	0	1	0
Apodachlya	0	0	0	1	0	0	0	1	0
Asterochloris	1	0	0	0	0	1	0	0	0
Aurigamonas	0	0	0	0	1	1	0	0	0
Batrachospermum	0	0	1	0	0	1	0	0	0
Betaphycus	0	0	0	1	0	0	0	1	0
Cephaleuros	1	0	0	0	0	1	0	0	0
Chlorococcaceae	0	0	0	0	0	1	0	1	0
Chloroidium	0	0	0	0	0	1	0	1	0
Cliftonaea	0	1	0	0	0	0	0	1	0
Clydonella	0	0	0	0	0	1	0	1	0
Colponema	0	0	0	0	0	1	0	1	0
Cymbomonas	0	0	1	0	0	1	0	0	0
Dasya	0	1	0	0	0	0	0	1	0
Dasycladus	1	1	0	0	0	0	0	0	0
Dichotomaria	0	1	0	0	0	0	0	1	0
Dicloster	0	0	0	0	0	1	0	1	0
Digenea	1	1	0	0	0	0	0	0	0
Dinobryon	0	0	0	0	0	1	0	1	0
Eustigmatos	0	1	0	0	0	0	0	1	0
Fibrocapsa	0	1	0	0	0	0	0	1	0
Focus	0	0	0	0	0	0	0	2	0
Fritschiella	0	1	0	1	0	0	0	0	0
Galaxaura	0	1	0	0	0	1	0	0	0
Gefionella	0	1	0	0	0	0	0	0	1
Golenkiniaceae	1	0	0	0	0	0	0	1	0
Hematodinium	0	1	0	0	0	0	0	1	0
Hyperamoeba	0	0	0	0	0	0	0	1	1

Hypnea	0	0	1	0	0	1	0	0	0
Labyrinthula	0	1	0	0	0	0	0	1	0
Laurencia	0	0	0	0	0	0	0	2	0
Lepidodinium	1	0	0	0	0	1	0	0	0
Liagora	1	1	0	0	0	0	0	0	0
Margalefidinium	1	0	0	0	0	1	0	0	0
Membranoptera	0	0	1	0	0	0	0	1	0
Neogoniolithon	0	1	0	0	0	0	0	1	0
Ophidocladus	0	0	0	0	0	1	0	1	0
Ophirina	1	0	0	0	0	0	0	0	1
Osmundaria	1	0	1	0	0	0	0	0	0
Oxymonadida	1	0	1	0	0	0	0	0	0
Oxyrrhis	0	0	0	0	0	1	0	1	0
Palmellopsis	0	0	0	0	0	1	0	1	0
Palmophyllum	1	0	0	0	0	1	0	0	0
Phaeoschizochlamys	0	0	0	0	0	0	0	2	0
Phytocercomonas	0	1	0	0	0	0	0	1	0
Picochlorum	0	0	0	0	0	1	0	1	0
Pleonosporium	0	1	1	0	0	0	0	0	0
Plumaria	0	0	0	0	1	0	0	1	0
Polymyxa	0	0	0	0	0	1	0	1	0
Pontisma	0	0	0	0	0	0	0	2	0
Protosiphon	0	1	1	0	0	0	0	0	0
Pterosiphonieae	0	0	2	0	0	0	0	0	0
Pterosperma	0	0	0	1	1	0	0	0	0
Pyrenomonas	0	0	0	0	0	0	0	1	1
Rhodogorgon	0	0	0	0	0	0	0	1	1
Schizomeris	0	1	0	0	0	1	0	0	0
Scotinosphaera	0	0	1	0	0	0	0	1	0
Sonderella	0	1	0	0	0	0	0	1	0
Spongomonas	0	0	0	0	0	1	1	0	0
Stachyamoeba	0	0	0	0	1	0	0	1	0
Stichococcus	0	0	0	1	0	0	0	1	0
Suigetsumonas clinomig	1	0	0	0	0	0	0	1	0
Telaepolella	1	1	0	0	0	0	0	0	0
Thaumatella	0	0	1	0	1	0	0	0	0
Thecamoeba	1	1	0	0	0	0	0	0	0
Thraustochytrium	0	0	0	1	0	0	0	1	0
Treubaria	0	0	0	0	0	1	0	1	0
Trochiscia	0	1	0	0	0	0	0	1	0
Unruhdinium	0	1	1	0	0	0	0	0	0
Vertebrata	0	0	1	0	0	1	0	0	0
Voromonas	0	1	0	0	0	0	1	0	0
Yamadaella	1	0	1	0	0	0	0	0	0

Acavomonas	1	0	0	0	0	0	0	0	0
Acrothesaurum	0	1	0	0	0	0	0	0	0
Allogromia	0	0	0	0	0	0	0	1	0
Amorphochlora	0	1	0	0	0	0	0	0	0
Antithamnion	0	0	0	1	0	0	0	0	0
Antithamnionella	0	0	0	0	0	0	0	1	0
Apoglossum	0	0	0	0	0	0	0	1	0
Aurantiochytrium	0	0	0	0	0	0	0	1	0
Aurearena	0	0	0	0	0	0	0	1	0
Ballia	0	0	0	0	0	1	0	0	0
Bangiopsis	0	0	0	0	0	0	0	1	0
Blattamonas	0	0	0	0	0	0	0	0	1
Blidingia	0	0	0	0	0	1	0	0	0
Brachidinium	1	0	0	0	0	0	0	0	0
Bryothamnion	1	0	0	0	0	0	0	0	0
Caulacanthus	0	0	0	0	0	0	0	1	0
Ceramium	0	0	0	0	0	1	0	0	0
Chaetomorpha	0	0	0	0	1	0	0	0	0
Chaetophoropsis	0	1	0	0	0	0	0	0	0
Chlorarachnion	0	0	0	0	0	1	0	0	0
Chlorochytriaceae	0	0	0	0	0	0	0	1	0
Chlorosarcinopsis	0	1	0	0	0	0	0	0	0
Choreocolax	0	0	0	0	0	0	0	1	0
Chromerida	0	0	0	0	0	1	0	0	0
Chromulina	0	1	0	0	0	0	0	0	0
Chrysamoeba	0	1	0	0	0	0	0	0	0
Chrysoparadoxa	0	0	0	0	0	1	0	0	0
Chrysotila	0	0	0	0	0	0	0	1	0
Coeloseira	1	0	0	0	0	0	0	0	0
Corythionella	0	0	0	0	0	0	0	1	0
Crassiphycus	0	0	0	0	0	0	0	1	0
Creneis	0	1	0	0	0	0	0	0	0
Dasyclonium	0	0	0	0	0	1	0	0	0
Dermonema	0	0	1	0	0	0	0	0	0
Dictyocha	0	0	0	0	0	0	0	1	0
Dimastigella	0	0	0	0	0	0	1	0	0
Diplopsalis	0	0	0	0	0	0	0	1	0
Dipterocladia	0	1	0	0	0	0	0	0	0
Dipterosiphonia	0	0	0	0	0	0	0	1	0
Enteromonas	0	1	0	0	0	0	0	0	0
Epipyxis	0	0	0	0	0	0	0	1	0
Ergobibamus	0	0	0	0	0	0	0	0	1
Eucheuma	1	0	0	0	0	0	0	0	0
Flabellulidae	1	0	0	0	0	0	0	0	0

Florenciella	0	0	0	0	0	0	0	1	0
Glossomastix	0	0	0	0	0	0	0	1	0
Griffithsia	0	0	0	0	0	0	0	1	0
Gromia oviformis	1	0	0	0	0	0	0	0	0
Gymnochlora	0	0	1	0	0	0	0	0	0
Haliphthoros	0	0	0	0	0	0	0	1	0
Halosaccion	0	0	0	0	0	0	0	1	0
Haptoglossa	1	0	0	0	0	0	0	0	0
Hazenia	0	0	0	0	0	0	0	1	0
Helminthora	0	0	0	0	0	0	0	1	0
Herposiphonia	1	0	0	0	0	0	0	0	0
Hicanonectes	0	0	0	0	0	0	0	0	1
Hommersandiophycus	0	0	1	0	0	0	0	0	0
Hormosira	0	0	0	0	0	0	0	1	0
Hyaloperonospora	0	0	0	0	0	0	0	1	0
Ishige	0	0	0	0	0	0	0	1	0
Kumanoa	0	0	1	0	0	0	0	0	0
Lagenidium	0	1	0	0	0	0	0	0	0
Leptochlorella	1	0	0	0	0	0	0	0	0
Massisteria	1	0	0	0	0	0	0	0	0
Miliammina	0	0	0	0	0	0	0	1	0
Monocystis	1	0	0	0	0	0	0	0	0
Montagnia	0	0	0	0	0	0	0	1	0
Nannochloris	0	0	0	0	0	0	0	1	0
Nemalion	0	1	0	0	0	0	0	0	0
Neomeris	0	0	0	1	0	0	0	0	0
Neoporphyra	0	0	0	0	0	1	0	0	0
Nitophyllum	0	0	0	0	0	0	0	1	0
Ochlochaete	0	0	0	0	0	0	0	1	0
Opalina	1	0	0	0	0	0	0	0	0
Osmundea	0	1	0	0	0	0	0	0	0
Parabodo caudatus	1	0	0	0	0	0	0	0	0
Paradoxia	0	0	0	0	0	0	0	1	0
Paraeustigmatos	0	0	1	0	0	0	0	0	0
Percolomonas	0	1	0	0	0	0	0	0	0
Phacotaceae	0	0	0	0	0	0	0	1	0
Phaeobotrys	0	0	0	0	0	0	0	1	0
Phaeocystis	0	0	0	0	0	0	0	0	1
Phaeosaccion	0	0	0	0	0	1	0	0	0
Pharyngomonas	0	0	0	0	0	0	0	0	1
Phytopythium	1	0	0	0	0	0	0	0	0
Pinguiococcus	0	0	0	0	0	0	0	1	0
Porphyropsis	0	0	1	0	0	0	0	0	0
Porphyrostromium	0	1	0	0	0	0	0	0	0

Pseudoperonospora	0	0	0	0	0	0	0	1	0
Pseudotetraedriella	0	0	0	0	1	0	0	0	0
Pseudulvella	1	0	0	0	0	0	0	0	0
Pyrodinium	0	0	1	0	0	0	0	0	0
Pyropia	0	0	0	0	0	0	0	1	0
Rhodomela	0	0	0	0	0	1	0	0	0
Rhodymenia	0	0	0	0	0	0	0	1	0
Rhynchomonas	0	0	0	0	0	1	0	0	0
Ripella	0	0	0	0	0	0	0	1	0
Sarcinofilum	0	0	0	0	0	0	0	1	0
Sawyeria	0	0	0	1	0	0	0	0	0
Schizochytrium	0	0	0	0	0	0	0	1	0
Schizymenia	0	1	0	0	0	0	0	0	0
Sclerospora	0	0	0	0	0	1	0	0	0
Sebdenia	0	0	0	0	0	1	0	0	0
Segregatospumella	1	0	0	0	0	0	0	0	0
Semnocarpa	1	0	0	0	0	0	0	0	0
Spiniferodinium	1	0	0	0	0	0	0	0	0
Stylonema	0	0	1	0	0	0	0	0	0
Symphyocladiella	1	0	0	0	0	0	0	0	0
Tetratrichomonas	0	1	0	0	0	0	0	0	0
Thuretia	0	0	0	0	0	1	0	0	0
Tolypiocladia	0	1	0	0	0	0	0	0	0
Trichomitus	0	0	0	0	0	0	0	1	0
Tupiella	0	1	0	0	0	0	0	0	0
Ulothrix	0	0	0	0	0	0	0	1	0
unclassified Thraustoch	0	0	0	0	0	0	0	1	0
Willea	0	1	0	0	0	0	0	0	0
Unknown	149	162	229	71	76	194	45	252	750

Supplementary File SF3. Rarefaction Analysis

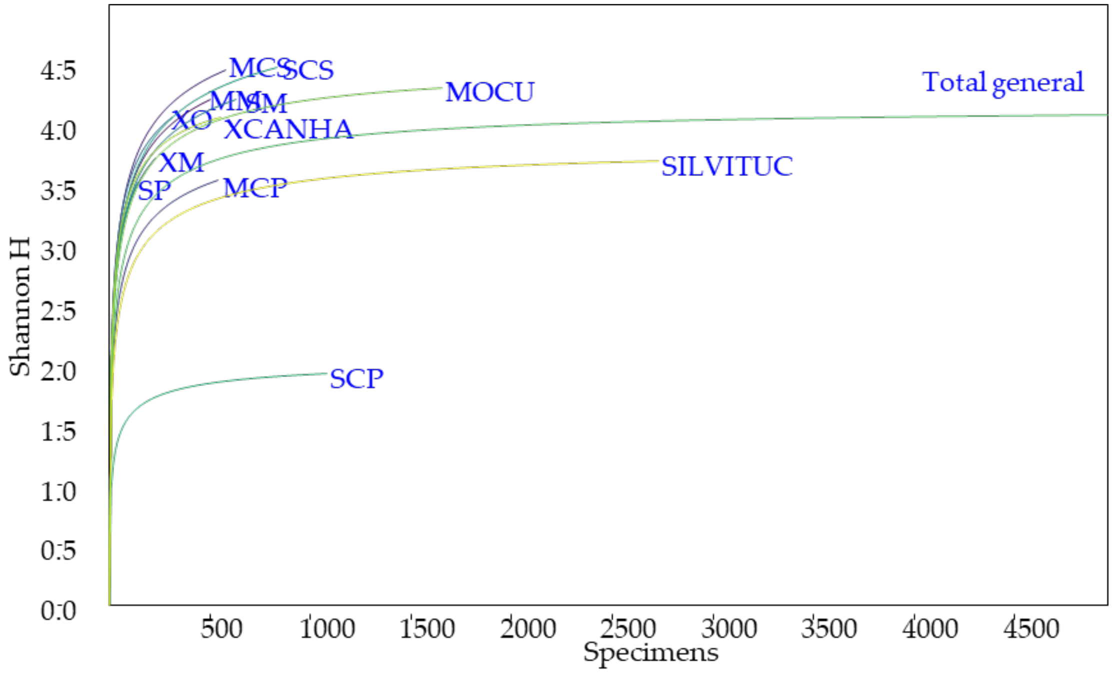

Supplementary File SF4. SIMPER Analysis

Taxon	Av. dissim	Contrib. %	Cumulative %	Mean Mocu	Mean Xcanha	Mean Silvituc
Theileria	1.563	2.854	2.854	3.67	2	14.5
Euglenophyceae	1.403	2.563	5.417	9	3.5	10.8
Hydrodictyon	1.084	1.979	7.396	8	3.5	8
Desmodesmus	1.042	1.902	9.299	6.67	9	8.25
Pseudochloris	0.7909	1.444	10.74	7	0	0.5
Volvocaceae	0.7634	1.394	12.14	5.67	8	6.25
Nannochloropsis	0.7627	1.393	13.53	7.67	7.5	3.25
Chlorella	0.7059	1.289	14.82	5.67	8	8
Ptilothamnion	0.6576	1.201	16.02	0.333	0	5.25
Cryptomonas	0.6557	1.197	17.22	6.33	1.5	6.25
Symbiodinium	0.5848	1.068	18.29	11	8.5	6.5
Ankistrodesmus	0.5029	0.9184	19.2	3	6	5.25
Vischeria	0.4487	0.8194	20.02	4.33	2	2.5
Synura	0.4441	0.8111	20.83	3	1.5	3
Eustigmatophyceae sp.	0.4196	0.7663	21.6	5.33	4.5	3.25
Chroomonas	0.4182	0.7638	22.36	4.33	1	2.5
Thraustotheca	0.384	0.7012	23.07	1	0.5	3.75
Haramonas	0.3826	0.6986	23.76	0.333	0	3
Prorocentrum	0.3719	0.6791	24.44	3.67	0.5	1.5
Alexandrium	0.3545	0.6474	25.09	3.33	0	1.25
Cyanophora	0.3391	0.6192	25.71	2.33	0	2.75
Acytosteliaceae	0.3338	0.6096	26.32	6.33	4	3.25
Trebouxia	0.3303	0.6031	26.92	3.33	2	1.75
Ulva	0.3046	0.5563	27.48	2	2	0.75
Porphyridium	0.3027	0.5529	28.03	2	1	2.75
Elliptochloris	0.2972	0.5428	28.57	2.33	0	0.5
Cyanidiaceae	0.2971	0.5425	29.12	0.667	0	2.25
Kappaphycus	0.2924	0.5341	29.65	0.333	0	2.25
Micromonas	0.2841	0.5189	30.17	3.33	3	4.75
Amphidinium	0.2623	0.4791	30.65	2.67	0	1.5
Melanthalia	0.2618	0.4781	31.13	0	0	2.25
Auxenochlorella	0.253	0.462	31.59	2.67	3.5	2.5
Dunaliellaceae	0.2478	0.4526	32.04	2	2.5	1.5
Chloropicon	0.2469	0.4508	32.49	2.33	1.5	2
Glaucocystis	0.2371	0.4331	32.93	1.33	0	2.25
Ochromonas	0.2362	0.4314	33.36	1.67	1	1.5
Coccomyxa	0.233	0.4255	33.78	2.33	3	2
Wildemania	0.2318	0.4233	34.21	0	0	2
Mastigamoebidae	0.2276	0.4156	34.62	0	0	2
Porolithon	0.2276	0.4156	35.04	0	0	2

Heterocapsa	0.2276	0.4156	35.45	2.33	0.5	0.5
Oedogonium	0.2247	0.4103	35.86	1.33	0.5	1.5
Tetraedron	0.2239	0.4089	36.27	0.667	1	1.75
Thaumatomonas	0.2233	0.4078	36.68	0	0	2
Reclinomonas	0.2215	0.4046	37.08	0.667	0.5	2
Nephroselmis	0.2214	0.4044	37.49	2	0.5	1.75
Scinaia	0.2186	0.3991	37.89	0.333	0	1.75
Quadricoccopsis	0.2171	0.3965	38.28	2	0.5	1
Diacronema	0.2143	0.3913	38.68	3	2	2
Neocystis	0.2137	0.3903	39.07	1.67	0.5	1.25
Azadinium	0.2118	0.3867	39.45	2	0	0
Nusuttodinium	0.2099	0.3833	39.84	2	0	0.5
Chattonella	0.2091	0.3819	40.22	1	0	1.75
Colpomenia	0.2086	0.3809	40.6	1.33	0	1
Dictyopteris	0.2077	0.3792	40.98	0.333	0	2
Pfiesteria	0.2072	0.3784	41.36	2	0	0.5
Ectocarpus	0.201	0.3671	41.72	2	3	2
Bracteacoccus	0.1983	0.3622	42.09	0.333	0.5	1.75
Vannella	0.1934	0.3531	42.44	0	0	1.75
Mallomonas	0.1919	0.3504	42.79	1	0	1.5
Emiliania	0.1916	0.35	43.14	2	1	2.25
Gracilaria	0.1906	0.348	43.49	0	0.5	1.5
Jenufa	0.1861	0.3399	43.83	1	0	1.25
Uroglenopsis	0.1852	0.3382	44.17	1.67	0	0
Proteomonas	0.1841	0.3363	44.5	1.33	1	1.5
Rhodomonas	0.1755	0.3204	44.82	1	0	1.5
Helicosporidium	0.174	0.3178	45.14	1.33	1.5	0.75
Sargassococcus	0.1723	0.3147	45.45	0.333	0	1.25
Globisporangium	0.1706	0.3115	45.77	2.33	1	0.75
Pedinomonas	0.1701	0.3107	46.08	1.33	0.5	1
Paramoeba	0.1633	0.2983	46.38	0	0	1.5
Chloroparvula	0.1632	0.298	46.67	1	0	1
Botryococcus	0.1605	0.2932	46.97	0.667	1	1.25
Spironucleus	0.1601	0.2924	47.26	0.333	0	1.25
Characiopsis	0.1586	0.2896	47.55	1.33	0.5	0.75
Peridinium	0.1585	0.2894	47.84	1	0	1
Eremosphaera	0.1568	0.2864	48.12	1	1	0.75
Trebouxiophyceae sp.	0.1542	0.2817	48.41	1	1	1
Spumella	0.151	0.2757	48.68	0.333	1	1
Erythrotrichia	0.147	0.2685	48.95	1.33	0	0.5
Herpetomonas	0.1468	0.2681	49.22	0.667	0	1.25
Karenia	0.1462	0.267	49.49	1.33	0	0.25
Melobesioideae	0.1457	0.2661	49.75	1.67	1	0.5
Microchloropsis	0.1457	0.266	50.02	1.67	2	1.5

Histiona	0.1456	0.266	50.28	1.67	0.5	1
Chlamydomonadales incertae sedis	0.1439	0.2628	50.55	1	1	1.5
Ochrosphaera	0.1434	0.2619	50.81	0.667	0	1.25
Amoeba	0.1431	0.2614	51.07	0.667	0	1
Uronema	0.1419	0.2592	51.33	1.33	0	0.25
Agarophyton	0.1408	0.2571	51.59	0.667	1	1
Tripos	0.1396	0.255	51.84	1.33	0	0.5
Vacuolaria	0.1396	0.255	52.1	1.33	0	0.5
Stichogloea	0.139	0.2539	52.35	0.667	0	0.75
Koliella	0.1388	0.2535	52.6	1.33	0.5	0.75
Ostreococcus	0.1382	0.2524	52.86	3	3	2
Cyanidioschyzon	0.136	0.2484	53.1	1.33	0.5	1.25
Ahnfeltia	0.1358	0.2481	53.35	1.33	1	1.5
Prymnesium	0.1356	0.2476	53.6	0	0.5	1
Jakoba	0.1346	0.2458	53.85	0.667	0	1
Cyanidiococcus	0.134	0.2447	54.09	2	2	1
Tritrichomonas	0.1317	0.2404	54.33	1.33	0	1
Marophrys	0.1312	0.2397	54.57	0.667	0	1
Carpediemonas	0.1312	0.2396	54.81	1.33	1.5	1.25
Goniomonas	0.1308	0.2388	55.05	0.333	0	1.25
Corallinoideae	0.1305	0.2383	55.29	0.667	0	1
Choricystis	0.1301	0.2376	55.52	0.667	1.5	1.25
Pterocladia	0.1297	0.2368	55.76	0	0	1
Bodo	0.1295	0.2364	56	1.67	1	1.5
Peronospora	0.1243	0.2269	56.22	1	1.5	0.5
Giardia	0.1233	0.2252	56.45	1	0	1
Microspora	0.122	0.2227	56.67	0.333	1	0.75
Tsunamia	0.1219	0.2226	56.89	0.667	0	1
Microthamnion	0.1214	0.2218	57.12	1	0	0.5
Pseudopedinella	0.12	0.2192	57.34	1	0.5	0.25
Hepatocystis	0.1177	0.215	57.55	0.667	1.5	1
Spongospora	0.1159	0.2117	57.76	0.333	0	1
Gracilariopsis	0.1142	0.2085	57.97	1.67	1.5	1.25
Prasinococcus	0.1134	0.2071	58.18	0.667	0	1
Crouania	0.1133	0.2069	58.38	1	0	0.5
Prototheca	0.1123	0.205	58.59	0.667	0	0.75
Pavlova	0.1116	0.2039	58.79	0	0	1
Ovalopodium	0.1116	0.2039	59	0	0	1
Balamuthiidae	0.1116	0.2039	59.2	0	0	1
Pseudoerythrocladia	0.1116	0.2039	59.41	0	0	1
Pseudellipsoidion	0.1112	0.2031	59.61	1	0.5	0.75
Pyramimonas	0.1104	0.2016	59.81	0.333	0.5	0.75
Tsukubamonas	0.1088	0.1988	60.01	0.667	0	1

Poteriospumella	0.1079	0.1971	60.21	1	0	0.25
Haematococcaceae	0.106	0.1936	60.4	1	1	1
Breviolum	0.1059	0.1934	60.59	1	0	0
Akashiwo	0.1059	0.1934	60.79	1	0	0
Kryptoperidinium	0.1059	0.1934	60.98	1	0	0
Martensia	0.1059	0.1934	61.17	1	0	0
Peridiniopsis	0.1059	0.1934	61.37	1	0	0
Flintiella	0.1059	0.1934	61.56	1	0	0
Pelagodinium	0.1059	0.1934	61.75	1	0	0
Perkinsela	0.1057	0.1931	61.95	1.33	0.5	1.25
Plocamium	0.1055	0.1927	62.14	0	0.5	0.75
Hildenbrandia	0.1049	0.1916	62.33	0.333	0	1
Thorea	0.1046	0.191	62.52	1	0.5	1
Guillardia	0.1046	0.191	62.71	2	1.5	2
Apusomonas	0.1038	0.1896	62.9	1	0	0.25
Acetabularia	0.1037	0.1893	63.09	0.667	0.5	0.25
Protodinium	0.1036	0.1892	63.28	1	0	0.25
Protoceratium	0.1036	0.1892	63.47	1	0	0.25
Dinophysis	0.1036	0.1892	63.66	1	0	0.25
Chromera	0.1036	0.1892	63.85	1	0	0.25
Karlodinium	0.1036	0.1892	64.04	1	0	0.25
Crypthecodinium	0.1036	0.1892	64.23	1	0	0.25
Cladocopium	0.1036	0.1892	64.42	1	0	0.25
Malawimonas	0.1031	0.1883	64.6	0	0	1
Kuetzingia	0.1031	0.1882	64.79	1	0.5	0.25
Andalucia	0.1029	0.1879	64.98	0.667	0.5	1.25
Oltmannsiellopsis	0.1027	0.1875	65.17	0.333	1	0.25
Cyanoptyche	0.1015	0.1853	65.35	1	0	0.5
Pyrocystis	0.1013	0.185	65.54	1	0	0.5
Renouxia	0.1013	0.185	65.72	1	0	0.5
Lingulodinium	0.1013	0.185	65.91	1	0	0.5
Durinskia	0.1013	0.185	66.09	1	0	0.5
Kipferlia	0.0997	0.1821	66.27	1	0	1
Pterosperma	0.0997	0.1821	66.46	0	1	0
Acrasis	0.0997	0.1821	66.64	0	1	0
Pedospumella	0.09927	0.1813	66.82	0.333	0.5	0.75
Telonemida	0.09921	0.1812	67	1	0	0.75
Schizocladia	0.09921	0.1812	67.18	1	0	0.75
Chrysochromulina	0.09894	0.1807	67.36	1	1	1.5
Compsopogon	0.0976	0.1782	67.54	0.667	1	0.25
Cyanidium	0.09749	0.178	67.72	0.667	0	0.5
Chromochloris	0.09612	0.1755	67.9	0.333	1	0.5
Monomastix	0.09609	0.1755	68.07	1	0.5	1
Scrippsiella	0.09585	0.175	68.25	1	0	0

Apophlaea	0.09555	0.1745	68.42	0.667	0.5	0
Vermamoeba	0.09549	0.1744	68.59	0.667	0	0.5
Lobosphaera	0.09549	0.1744	68.77	0.333	1	0.5
Cafileria marina	0.09545	0.1743	68.94	0	0	0.75
Paracercomonas	0.09545	0.1743	69.12	0	0	0.75
Tetraselmis	0.09507	0.1736	69.29	0.333	1	0.5
Chordaria	0.09476	0.1731	69.46	0	0.5	0.75
Fusochloris	0.09476	0.1731	69.64	0	0.5	0.75
Isochrysis	0.09411	0.1719	69.81	0.667	0.5	0
Grateloupia	0.09356	0.1709	69.98	0	0.5	0.75
Picocystis	0.09323	0.1703	70.15	0.667	0	0.75
Storeatula	0.09323	0.1703	70.32	0.667	0	0.75
Pelagomonas	0.09321	0.1702	70.49	1.33	1	1.25
Tetrabaenaceae	0.09247	0.1689	70.66	1	1	0.75
Rhodochaete	0.09185	0.1677	70.83	0.667	0	0.75
prasinophyte sp.	0.09185	0.1677	71	0.667	0	0.75
Gloeochaete	0.09185	0.1677	71.16	0.667	0	0.75
Korotnevella	0.09075	0.1657	71.33	0.667	0	0.75
unclassified Cryptophyta	0.09023	0.1648	71.49	0	0.5	0.75
Schimmelmannia	0.08968	0.1638	71.66	1	0.5	0.5
Monocercomonoides	0.08968	0.1638	71.82	1	0.5	0.5
Gloiopeltis	0.08968	0.1638	71.98	1	0.5	0.5
Cardiosporidium	0.08968	0.1638	72.15	1	0.5	0.5
Gelidium	0.08886	0.1623	72.31	0.333	0	0.75
Leucocryptos	0.08808	0.1609	72.47	0.667	0	0.5
Scherffelia	0.08733	0.1595	72.63	0.667	1	0.5
Monodopsis	0.08733	0.1595	72.79	0.667	1	0.5
Placidia	0.08671	0.1584	72.95	0.667	0.5	0.25
Bigelowiella	0.08615	0.1573	73.11	0.333	1	0.5
Aureoumbra	0.08592	0.1569	73.26	0.667	0.5	0.5
Polypodochrysis	0.08591	0.1569	73.42	0	0	0.75
Heterosigma	0.08538	0.1559	73.58	0.667	0.5	0.5
Paratrypanosoma	0.08512	0.1554	73.73	0.667	0.5	0.25
Phalacroma	0.08489	0.155	73.89	0.333	0	0.5
Protosteliaceae	0.08477	0.1548	74.04	0.333	0	0.75
Chlorosarcina	0.08471	0.1547	74.2	0.333	0	0.75
Polysiphonia	0.08471	0.1547	74.35	0.333	0	0.75
Teleaulax	0.08467	0.1546	74.51	0.667	0	0.5
Boldia	0.08467	0.1546	74.66	0.667	0	0.5
Moramonas	0.08467	0.1546	74.81	0.667	0	0.5
Olisthodiscus	0.08413	0.1536	74.97	0.667	0.5	0.5
Palpitomonas	0.08339	0.1523	75.12	0.333	0	0.75
Hemiarma	0.08339	0.1523	75.27	0.333	0	0.75
Triparma	0.08329	0.1521	75.43	0.667	0.5	0.5

Myrmecia	0.08329	0.1521	75.58	0.667	0.5	0.5
Prasiola	0.08329	0.1521	75.73	0.667	0.5	0.5
Pseudochlorella	0.08329	0.1521	75.88	0.667	0.5	0.5
Pleurastraceae	0.08329	0.1521	76.03	0.667	0.5	0.5
Ignatiu	0.08327	0.1521	76.19	0.667	0.5	0.5
Cryptocaryon	0.08313	0.1518	76.34	0.667	0	0.5
Neopyropia	0.08313	0.1518	76.49	0.667	0	0.5
Pleurostomum	0.0831	0.1518	76.64	0.333	0.5	0.5
Mesophyllum	0.0816	0.149	76.79	0	0	0.75
Symbiochloris	0.08156	0.149	76.94	0.333	0.5	0.5
Poterioochromonas	0.08134	0.1486	77.09	0.667	0	0.5
Parachlorella	0.08134	0.1486	77.24	0.667	0	0.5
Gonyostomum	0.08134	0.1486	77.38	0.667	0	0.5
Dictyochloris	0.08134	0.1486	77.53	0.667	0	0.5
Balbiania	0.08134	0.1486	77.68	0.667	0	0.5
unclassified Chlamydomonadales	0.08134	0.1486	77.83	0.667	0	0.5
Prasiolopsis	0.08035	0.1467	77.98	0.667	0	0.25
Chroodactylon	0.08035	0.1467	78.12	0.667	0	0.25
Euglypha	0.08015	0.1464	78.27	0.333	0	0.5
Bulboplastis	0.07988	0.1459	78.42	0.667	0.5	0.75
Dicranema	0.07937	0.1449	78.56	0.333	0.5	0.25
Halochlorococcum	0.07908	0.1444	78.71	0.667	0	0.25
Marvania	0.07879	0.1439	78.85	0.333	0.5	0.5
Taenioma	0.07843	0.1432	78.99	0	0.5	0.5
Nephromyces	0.0783	0.143	79.14	0.333	0.5	0.25
Geminella	0.0783	0.143	79.28	0.333	0.5	0.25
Lacrimia	0.07814	0.1427	79.42	0.333	0.5	0.25
Haptophyceae sp. NIES-3900	0.07814	0.1427	79.56	0.333	0.5	0.25
Polarella	0.07812	0.1427	79.71	1	1	0.5
Nothophytophthora	0.07812	0.1427	79.85	1	1	0.5
Trichomonas	0.07812	0.1427	79.99	1	1	1
Histomonas	0.07812	0.1427	80.13	1	1	1
Trachydiscus	0.07812	0.1427	80.28	1	1	0.5
Bostrychia	0.07798	0.1424	80.42	0.667	0	0.25
Andersenia	0.07798	0.1424	80.56	0.667	0	0.25
Leptophrys	0.07786	0.1422	80.7	0	0.5	0.5
Blepharisma	0.07785	0.1422	80.85	0.333	0	0.5
Pseudomuriella	0.07774	0.142	80.99	0.333	0.5	0.5
Marsupiomonas	0.07741	0.1414	81.13	0	0	0.75
Vitrella	0.07698	0.1406	81.27	1.67	1	1.25
Pterosiphonieae	0.07663	0.1399	81.41	0.667	0	0
Acrosorium	0.07596	0.1387	81.55	0.667	0	0.25
Seculamonas	0.07596	0.1387	81.69	0.667	0	0.25
Paravannella	0.07596	0.1387	81.83	0.667	0	0.25

Madagascaria	0.07596	0.1387	81.97	0.667	0	0.25
Xylochloris	0.07564	0.1381	82.1	0.333	0.5	0.25
Watanabea	0.07564	0.1381	82.24	0.333	0.5	0.25
Picozoa	0.0755	0.1379	82.38	0	0.5	0.5
Yamadaella	0.07394	0.135	82.51	0.667	0	0
Oxymonadida	0.07394	0.135	82.65	0.667	0	0
Osmundaria	0.07394	0.135	82.78	0.667	0	0
Spongomonas	0.07392	0.135	82.92	0	0	0.5
Stygiella	0.07392	0.135	83.05	1	1	0.5
Lithomelissa	0.07364	0.1345	83.19	0.667	0	0.25
Parietochloris	0.07323	0.1337	83.32	0	0.5	0.5
Gloeotilopsis	0.07323	0.1337	83.46	0	0.5	0.5
Fritschiella	0.07284	0.133	83.59	0.333	0.5	0
Jaagichlorella	0.07283	0.133	83.72	0.333	0.5	0.25
unc.	0.07273	0.1328	83.86	1	0.5	0.75
Thaumatella	0.07089	0.1295	83.98	0.333	0.5	0
Unruhdinium	0.07026	0.1283	84.11	0.667	0	0
Protosiphon	0.07026	0.1283	84.24	0.667	0	0
Pleonosporium	0.07026	0.1283	84.37	0.667	0	0
Palmaria	0.07	0.1278	84.5	0.333	0	0.5
Hemiselmis	0.07	0.1278	84.63	0.333	0	0.5
Caloglossa	0.0689	0.1258	84.75	0	0	0.75
Cavenderiaceae	0.06869	0.1254	84.88	1	0.5	0.75
Chondrus	0.06869	0.1254	85	1	0.5	0.75
Thecamoeba	0.06757	0.1234	85.13	0.667	0	0
Telaepolella	0.06757	0.1234	85.25	0.667	0	0
Liagora	0.06757	0.1234	85.37	0.667	0	0
Digenea	0.06757	0.1234	85.5	0.667	0	0
Dasycladus	0.06757	0.1234	85.62	0.667	0	0
Rhizochromulina	0.06733	0.123	85.74	0.333	0	0.5
Gambierdiscus	0.06733	0.123	85.86	0.333	0	0.5
Desmarestia	0.06733	0.123	85.99	0.333	0	0.5
Thecamonas	0.06624	0.121	86.11	1.33	1	1.5
Phalansterium	0.06624	0.121	86.23	0.333	0	0.5
Corynoplastis	0.06624	0.121	86.35	0.333	0	0.5
Voromonas	0.06607	0.1207	86.47	0.333	0	0.25
Adenoides	0.06554	0.1197	86.59	0.333	0	0.5
Toxarium	0.06554	0.1197	86.71	0.333	0	0.5
Rotundella	0.06554	0.1197	86.83	0.333	0	0.5
Neodangemannia	0.06554	0.1197	86.95	0.333	0	0.5
Edaphochlorella	0.06554	0.1197	87.07	0.333	0	0.5
Thraustochytrium	0.06504	0.1188	87.19	0	0.5	0.25
Stichococcus	0.06504	0.1188	87.31	0	0.5	0.25
Betaphycus	0.06504	0.1188	87.43	0	0.5	0.25

Apodachlya	0.06504	0.1188	87.54	0	0.5	0.25
Feldmannia	0.06459	0.118	87.66	0	0	0.75
Asparagopsis	0.06318	0.1154	87.78	0.333	0	0.5
Sticholonche	0.06318	0.1154	87.89	0.333	0	0.5
Neotessella	0.06318	0.1154	88.01	0.333	0	0.5
Chondria	0.06318	0.1154	88.12	0.333	0	0.5
Aurigamonas	0.06087	0.1112	88.24	0	0.5	0.25
Stachyamoeba	0.0585	0.1068	88.34	0	0.5	0.25
Plumaria	0.0585	0.1068	88.45	0	0.5	0.25
Plasmodiophora	0.05685	0.1038	88.55	0.667	1	0.75
Hondaea	0.05685	0.1038	88.66	0.667	1	0.75
Cafeteria	0.05685	0.1038	88.76	0.667	1	0.75
Gregarina	0.05685	0.1038	88.86	0.667	1	0.75
Reticulomyxa	0.05685	0.1038	88.97	0.667	1	0.75
Porphyra	0.05588	0.102	89.07	1	1	1
Ophirina	0.05485	0.1002	89.17	0.333	0	0.25
Vertebrata	0.05418	0.09894	89.27	0.333	0	0.25
Hypnea	0.05418	0.09894	89.37	0.333	0	0.25
Cymbomonas	0.05418	0.09894	89.47	0.333	0	0.25
Batrachospermum	0.05418	0.09894	89.57	0.333	0	0.25
Acrochaetium	0.05418	0.09894	89.67	0.333	0	0.25
Sawyeria	0.05356	0.09782	89.76	0	0.5	0
Neomeris	0.05356	0.09782	89.86	0	0.5	0
Antithamnion	0.05356	0.09782	89.96	0	0.5	0
Gefionella	0.05216	0.09526	90.05	0.333	0	0.25
Palmophyllum	0.05201	0.09499	90.15	0.333	0	0.25
Margalefidinium	0.05201	0.09499	90.24	0.333	0	0.25
Lepidodinium	0.05201	0.09499	90.34	0.333	0	0.25
Cephaleuros	0.05201	0.09499	90.43	0.333	0	0.25
Asterochloris	0.05201	0.09499	90.53	0.333	0	0.25
Pyrenomonas	0.05157	0.09417	90.62	0	0	0.5
Hyperamoeba	0.05157	0.09417	90.72	0	0	0.5
Rhodogorgon	0.05157	0.09417	90.81	0	0	0.5
Scotinosphaera	0.05146	0.09399	90.91	0.333	0	0.25
Membranoptera	0.05146	0.09399	91	0.333	0	0.25
Suigetsumonas clinomigrationis	0.04915	0.08976	91.09	0.333	0	0.25
Golenkiniaceae	0.04915	0.08976	91.18	0.333	0	0.25
Aphanochaete	0.04915	0.08976	91.27	0.333	0	0.25
Schizomeris	0.0491	0.08967	91.36	0.333	0	0.25
Galaxaura	0.0491	0.08967	91.45	0.333	0	0.25
Dimastigella	0.04808	0.08781	91.54	0	0	0.25
Treubaria	0.04737	0.08651	91.62	0	0	0.5
Polymyxa	0.04737	0.08651	91.71	0	0	0.5
Picochlorum	0.04737	0.08651	91.8	0	0	0.5

Palmellopsis	0.04737	0.08651	91.88	0	0	0.5
Oxyrrhis	0.04737	0.08651	91.97	0	0	0.5
Ophidocladus	0.04737	0.08651	92.05	0	0	0.5
Dinobryon	0.04737	0.08651	92.14	0	0	0.5
Dicloster	0.04737	0.08651	92.23	0	0	0.5
Colponema	0.04737	0.08651	92.31	0	0	0.5
Clydonella	0.04737	0.08651	92.4	0	0	0.5
Chloroidium	0.04737	0.08651	92.49	0	0	0.5
Chlorococcaceae	0.04737	0.08651	92.57	0	0	0.5
Pseudotetraedriella	0.04614	0.08426	92.66	0	0.5	0
Chaetomorpha	0.04614	0.08426	92.74	0	0.5	0
Trochiscia	0.04603	0.08405	92.83	0.333	0	0.25
Sonderella	0.04603	0.08405	92.91	0.333	0	0.25
Phytocercomonas	0.04603	0.08405	92.99	0.333	0	0.25
Neogoniolithon	0.04603	0.08405	93.08	0.333	0	0.25
Labyrinthula	0.04603	0.08405	93.16	0.333	0	0.25
Hematodinium	0.04603	0.08405	93.25	0.333	0	0.25
Fibrocapsa	0.04603	0.08405	93.33	0.333	0	0.25
Eustigmatos	0.04603	0.08405	93.41	0.333	0	0.25
Dichotomaria	0.04603	0.08405	93.5	0.333	0	0.25
Dasya	0.04603	0.08405	93.58	0.333	0	0.25
Cliftonaea	0.04603	0.08405	93.67	0.333	0	0.25
Pontisma	0.04306	0.07864	93.75	0	0	0.5
Phaeoschizochlamys	0.04306	0.07864	93.82	0	0	0.5
Laurencia	0.04306	0.07864	93.9	0	0	0.5
Focus	0.04306	0.07864	93.98	0	0	0.5
Paraeustigmatos	0.03831	0.06997	94.05	0.333	0	0
Pyrodinium	0.03831	0.06997	94.12	0.333	0	0
Porphyropsis	0.03831	0.06997	94.19	0.333	0	0
Kumanoa	0.03831	0.06997	94.26	0.333	0	0
Hommersandiophycus	0.03831	0.06997	94.33	0.333	0	0
Gymnochlora	0.03831	0.06997	94.4	0.333	0	0
Dermonema	0.03831	0.06997	94.47	0.333	0	0
Stylonema	0.03831	0.06997	94.54	0.333	0	0
Symphyocladiella	0.03562	0.06506	94.61	0.333	0	0
Spiniferodinium	0.03562	0.06506	94.67	0.333	0	0
Segregatospumella	0.03562	0.06506	94.74	0.333	0	0
Pseudulvella	0.03562	0.06506	94.8	0.333	0	0
Phytopythium	0.03562	0.06506	94.87	0.333	0	0
Parabodo caudatus	0.03562	0.06506	94.93	0.333	0	0
Opalina	0.03562	0.06506	95	0.333	0	0
Monocystis	0.03562	0.06506	95.06	0.333	0	0
Massisteria	0.03562	0.06506	95.13	0.333	0	0
Leptochlorella	0.03562	0.06506	95.19	0.333	0	0

Herposiphonia	0.03562	0.06506	95.26	0.333	0	0
Haptoglossa	0.03562	0.06506	95.32	0.333	0	0
Gromia oviformis	0.03562	0.06506	95.39	0.333	0	0
Flabellulidae	0.03562	0.06506	95.45	0.333	0	0
Eucheuma	0.03562	0.06506	95.52	0.333	0	0
Semnocarpa	0.03562	0.06506	95.58	0.333	0	0
Coeloseira	0.03562	0.06506	95.65	0.333	0	0
Bryothamnion	0.03562	0.06506	95.71	0.333	0	0
Brachidinium	0.03562	0.06506	95.78	0.333	0	0
Acavomonas	0.03562	0.06506	95.84	0.333	0	0
Willea	0.03195	0.05835	95.9	0.333	0	0
Tupiella	0.03195	0.05835	95.96	0.333	0	0
Tolypiocladia	0.03195	0.05835	96.02	0.333	0	0
Tetratrichomonas	0.03195	0.05835	96.08	0.333	0	0
Porphyrostromium	0.03195	0.05835	96.13	0.333	0	0
Percolomonas	0.03195	0.05835	96.19	0.333	0	0
Osmundea	0.03195	0.05835	96.25	0.333	0	0
Nemalion	0.03195	0.05835	96.31	0.333	0	0
Lagenidium	0.03195	0.05835	96.37	0.333	0	0
Enteromonas	0.03195	0.05835	96.43	0.333	0	0
Dipterocladia	0.03195	0.05835	96.48	0.333	0	0
Schizymenia	0.03195	0.05835	96.54	0.333	0	0
Creneis	0.03195	0.05835	96.6	0.333	0	0
Chrysamoeba	0.03195	0.05835	96.66	0.333	0	0
Chromulina	0.03195	0.05835	96.72	0.333	0	0
Chlorosarcinopsis	0.03195	0.05835	96.78	0.333	0	0
Chaetophoropsis	0.03195	0.05835	96.83	0.333	0	0
Amorphochlora	0.03195	0.05835	96.89	0.333	0	0
Acrothesaurum	0.03195	0.05835	96.95	0.333	0	0
Pharyngomonas	0.03004	0.05485	97.01	0	0	0.25
Phaeocystis	0.03004	0.05485	97.06	0	0	0.25
Hicanonectes	0.03004	0.05485	97.12	0	0	0.25
Streblomastix	0.03004	0.05485	97.17	1	1	0.75
Ergobibamus	0.03004	0.05485	97.23	0	0	0.25
Raperosteliaceae	0.03004	0.05485	97.28	1	1	0.75
Plasmopara	0.03004	0.05485	97.33	1	1	0.75
Blattamonas	0.03004	0.05485	97.39	0	0	0.25
Galdieria	0.03004	0.05485	97.44	1	1	0.75
Bathycoccus	0.03004	0.05485	97.5	1	1	0.75
Aureococcus	0.03004	0.05485	97.55	1	1	0.75
Achlya	0.03004	0.05485	97.61	1	1	0.75
Thuretia	0.02584	0.04719	97.66	0	0	0.25
Sebdenia	0.02584	0.04719	97.7	0	0	0.25
Sclerospora	0.02584	0.04719	97.75	0	0	0.25

Rhynchomonas	0.02584	0.04719	97.8	0	0	0.25
Rhodomela	0.02584	0.04719	97.85	0	0	0.25
Phaeosaccion	0.02584	0.04719	97.89	0	0	0.25
Neoporphyra	0.02584	0.04719	97.94	0	0	0.25
Dasyclonium	0.02584	0.04719	97.99	0	0	0.25
Chrysoparadoxa	0.02584	0.04719	98.03	0	0	0.25
Chromerida	0.02584	0.04719	98.08	0	0	0.25
Chlorarachnion	0.02584	0.04719	98.13	0	0	0.25
Ceramium	0.02584	0.04719	98.18	0	0	0.25
Blidingia	0.02584	0.04719	98.22	0	0	0.25
Ballia	0.02584	0.04719	98.27	0	0	0.25
unclassified Thraustochytriidae	0.02153	0.03932	98.31	0	0	0.25
Ulothrix	0.02153	0.03932	98.35	0	0	0.25
Trichomitus	0.02153	0.03932	98.39	0	0	0.25
Schizochytrium	0.02153	0.03932	98.43	0	0	0.25
Sarcinofilum	0.02153	0.03932	98.47	0	0	0.25
Ripella	0.02153	0.03932	98.51	0	0	0.25
Rhodymenia	0.02153	0.03932	98.55	0	0	0.25
Pyropia	0.02153	0.03932	98.58	0	0	0.25
Pseudoperonospora	0.02153	0.03932	98.62	0	0	0.25
Pinguiococcus	0.02153	0.03932	98.66	0	0	0.25
Phaeobotrys	0.02153	0.03932	98.7	0	0	0.25
Phacotaceae	0.02153	0.03932	98.74	0	0	0.25
Paradoxia	0.02153	0.03932	98.78	0	0	0.25
Ochlochaete	0.02153	0.03932	98.82	0	0	0.25
Nitophyllum	0.02153	0.03932	98.86	0	0	0.25
Nannochloris	0.02153	0.03932	98.9	0	0	0.25
Montagnia	0.02153	0.03932	98.94	0	0	0.25
Miliammina	0.02153	0.03932	98.98	0	0	0.25
Ishige	0.02153	0.03932	99.02	0	0	0.25
Hyaloperonospora	0.02153	0.03932	99.06	0	0	0.25
Hormosira	0.02153	0.03932	99.1	0	0	0.25
Helminthora	0.02153	0.03932	99.13	0	0	0.25
Hazenia	0.02153	0.03932	99.17	0	0	0.25
Halosaccion	0.02153	0.03932	99.21	0	0	0.25
Haliphthoros	0.02153	0.03932	99.25	0	0	0.25
Griffithsia	0.02153	0.03932	99.29	0	0	0.25
Glossomastix	0.02153	0.03932	99.33	0	0	0.25
Florenciella	0.02153	0.03932	99.37	0	0	0.25
Epipyxis	0.02153	0.03932	99.41	0	0	0.25
Dipterosiphonia	0.02153	0.03932	99.45	0	0	0.25
Diplopsalis	0.02153	0.03932	99.49	0	0	0.25
Dictyocha	0.02153	0.03932	99.53	0	0	0.25
Crassiphycus	0.02153	0.03932	99.57	0	0	0.25

Corythionella	0.02153	0.03932	99.61	0	0	0.25
Chrysotila	0.02153	0.03932	99.65	0	0	0.25
Choreocolax	0.02153	0.03932	99.69	0	0	0.25
Chlorochytriaceae	0.02153	0.03932	99.72	0	0	0.25
Caulacanthus	0.02153	0.03932	99.76	0	0	0.25
Bangiopsis	0.02153	0.03932	99.8	0	0	0.25
Aurearena	0.02153	0.03932	99.84	0	0	0.25
Aurantiochytrium	0.02153	0.03932	99.88	0	0	0.25
Apoglossum	0.02153	0.03932	99.92	0	0	0.25
Antithamnionella	0.02153	0.03932	99.96	0	0	0.25
Allogromia	0.02153	0.03932	100	0	0	0.25
Pycnococcus	0	0	100	1	1	1
Planoprotostelium	0	0	100	1	1	1
Pelomyxidae	0	0	100	1	1	1
Pedinophyceae sp.	0	0	100	1	1	1
